# SPAG5 interacts with CEP55 and exerts oncogenic activities via PI3K/AKT pathway in hepatocellular carcinoma

**DOI:** 10.1186/s12943-018-0872-3

**Published:** 2018-08-08

**Authors:** Yu-Feng Yang, Mei-Fang Zhang, Qiu-Hong Tian, Jia Fu, Xia Yang, Chris Zhiyi Zhang, Hong Yang

**Affiliations:** 1 Department of Pathology, Dongguan Third People’s Hospital, Dongguan, China; 2 Department of Pathology, Sun Yat-sen University Cancer Center, State Key Laboratory of Oncology in South China, Collaborative Innovation Center for Cancer Medicine, Guangzhou, 510060 Guangdong China; 3 Department of Oncology, First Affiliated Hospital of NanChang University, NanChang, 330006 Jiangxi China; 4 Department of Thoracic Oncology, Sun Yat-sen University Cancer Center, State Key Laboratory of Oncology in South China, Collaborative Innovation Center for Cancer Medicine, 651 Dongfeng Road East, Guangzhou, 510060 China

**Keywords:** SPAG5, CEP55, miR-363-3p, PI3K/AKT, Hepatocellular carcinoma

## Abstract

**Background:**

Deregulation of microtubules and centrosome integrity is response for the initiation and progression of human cancers. Sperm-associated antigen 5 (SPAG5) is essential for the spindle apparatus organization and chromosome segregation, but its role in hepatocellular carcinoma (HCC) remains undefined.

**Methods:**

The expression of SPAG5 in HCC were examined in a large cohort of patients by RT-PCR, western blot and IHC. The clinical significance of SPAG5 was next determined by statistical analyses. The biological function of SPAG5 in HCC and the underlying mechanisms were investigated, using in vitro and in vivo models.

**Results:**

Here, we demonstrated that SPAG5 exhibited pro-HCC activities via the activation of PI3K/AKT signaling pathway. SPAG5 expression was increased in HCC and correlated with poor outcomes in two independent cohorts containing 670 patients. High SPAG5 expression was associated with poor tumor differentiation, larger tumor size, advanced TNM stage, tumor vascular invasion and lymph node metastasis. In vitro and in vivo data showed that SPAG5 overexpression promoted tumor growth and metastasis, whereas SPAG5 knockdown led to the opposite phenotypes. SPAG5 interacted with centrosomal protein CEP55 to trigger the phosphorylation of AKT at Ser473. Inhibition of PI3K/AKT signaling markedly attenuated SPAG5-mediated cell growth. Furthermore, SPAG5 expression was suppressed by miR-363-3p which inhibited the activity of SPAG5 mRNA 3’UTR. Ectopic expression of SPAG5 partly abolished the miR-363-3p-caused cell cycle arrest and suppression of cell proliferation and migration.

**Conclusions:**

Collectively, these findings indicate that SPAG5 serves a promising prognostic factor in HCC and functions as an oncogene via CEP55-mediated PI3K/AKT pathway. The newly identified miR-363-3p/SPAG5/CEP55 axis may represent a potential therapeutic target for the clinical intervention of HCC.

**Electronic supplementary material:**

The online version of this article (10.1186/s12943-018-0872-3) contains supplementary material, which is available to authorized users.

## Background

Liver cancer represents one of the most frequent malignant diseases in China and the global [1, 2]. According to a histopathological perspective, more than 90% of liver cancer belongs to hepatocellular carcinoma (HCC). Over 450,000 HCC cases were newly diagnosed, and 420,000 HCC patients were dead in 2015 in China [1]. The mortality rate of HCC ranks the second most common cause of cancer-related death in men and the sixth in women. Unfortunately, the molecular mechanisms of HCC development and progression remain so far obscure. Previous literatures indicate that deregulation of genes involved in cell cycle regulation contributes to the hepatocarcinogenesis [3, 4]. Studies focus on the role of such genes may provide clinical significance to the management of HCC.

Sperm-associated antigen 5 (SPAG5, also known as Astrin and hMAP126), mapped to Ch17q11.2, was originally identified as a microtubule-associated protein that was associated with spindles throughout mitosis and localized to kinetochores of congressed chromosomes [5, 6]. SPAG5 contains a N-terminal globular domain and two predicted coiled-coil domains. SPAG5 has been demonstrated to interact with many proteins, such as Aurora-A [7], PLK1 [8] and GSK3β [9] to modulate the spindle apparatus organization and chromosome segregation, making SPAG5 essential in the cell growth. Centrosome instability and malformation of the spindle were observed in SPAG5-depleted cells [10]. Silencing of SPAG5 suppressed the growth of HeLa cells and resulted in the formation of multipolar and highly disordered spindles [11]. Overexpression of SPAG5 was reported in cervical cancer, pancreatic cancer and non-small-cell lung cancer [12–14]. Amplification or gain of the SPAG5 locus occurring in 10–19% of breast cancers was correlated with poor clinical outcome and adverse clinicopathological features [15]. Furthermore, the upregulation of SPAG5 affected the response of cancer cells to chemotherapy [12, 15]. These data strongly link SPAG5 to the progression of human cancers. However, the clinical significance of SPAG5 and its role in HCC are still unclear.

Using tissue microarray, and in vitro and in vivo models, we intended to examine the expression and clinical value of SPAG5 in HCC, and to explore the role of SPAG5 in HCC cell growth and the underlying mechanisms. Our data suggest SPAG5 serve as a potential prognostic factor and function as an oncogene via CEP55-mediated PI3K/AKT pathway in HCC.

## Methods

### Patients

Twenty-seven fresh HCC specimens were collected for determination of mRNA and protein levels of SPAG5 from Sun Yat-sen University Cancer Center (SYSUCC). A cohort of 298 paraffin-embedded HCC cases diagnosed between Jan 2012 to Dec 2013 at Dongguan Third People’s Hospital and SYSUCC was recruited. Another 93 HCC samples with venous metastases were obtained from SYSUCC. None of the patients had received radiotherapy or chemotherapy before surgery. All samples were anonymous. This project was approved by Institute Research Ethics Committee of the above two hospitals. The clinical implication of SPAG5 was further determined in The Cancer Genome Atlas (TCGA) dataset (http://www.cbioportal.org) and the Oncomine dataset (https://www.oncomine.org).

### Cell culture and transfection

HCC cell lines PLC8024, Huh7 and QGY-7703 were purchased from the Cell Resource Center, Chinese Academy of Science Committee (Shanghai, China). Cells were maintained in Dulbecco’s modified Eagle’s medium (DMEM) (Gibco, Gaithersburg, MD, USA) supplemented with 10% heat-inactivated fetal bovine serum (FBS, Hyclone, Logan, UT) in a humidified incubator at 37 °C and 5% CO_2_. The cells were transfected with SPAG5 overexpression vector or shRNAs by Lipofectamine 2000, according to the instruction, and then selected by G418 for 4 weeks to establish stable cells.

### Quantitative real-time polymerase chain reaction (qRT-PCR)

qRT-PCR was performed according to our previous study. The sequences of the PCR primers are as followings: SPAG5, forward: 5′- CATCTCACAGTGGGATAACTAATAAAC-3′ and reverse: 5′- CAGGGATAGGTGAAGCAAGGATA-3′; CEP55, forward: 5’-TGAAGAGAAAGACGTATTGAAACAA-3′ and reverse: 5′- ACTGTGGCTCCAAACTGCTT-3′; β-actin, forward: 5’-TGGCACCCAGCACAATGAA-3′ and reverse: 5’-CTAAGTCATAGTCCGCCTAGAAGCA-3′. The relative expression of SPAG5 and CEP55 was presented as –ΔCT value.

### Western blot

Equal amounts of protein (30 μg) were resolved by SDS-PAGE and then electrophoretically transferred onto PVDF membranes (Millipore, Bedford, MA). After blocked in 5% non-fat milk 1 h at room temperature, the membranes were incubated with appropriately diluted primary antibodies overnight at 4 °C. After washed twice with TBST, the blotted membranes were incubated with HRP-conjugated secondary antibody at 1:20000 dilutions for 1 h at room temperature. The membranes were visualized by the enhanced Phototope TM-HRP Detection Kit and exposed to Kodak medical X-ray processor (Carestream Health, USA). The primary antibodies are as followings: SPAG5, 1:500 (Sigma-Aldrich), CEP55 (1:1000, #81693, Cell Signaling Technology), AKT (1:1000, #2920, Cell Signaling Technology), p-AKT at Ser473 (1:1000, #4046, Cell Signaling Technology), ERK1/2 (1:1000, #4695, Cell Signaling Technology), p-ERK1/2 at Thr202/Tyr204 (1:1000, #4370, Cell Signaling Technology), E-Cadherin, β-Catenin, N-Cadherin, Vimentin, Fibronectin and MMP-2 (Epitomics, Burlingame, CA) and β-actin (1:1000, #3700, Cell Signaling Technology).

### Immunohistochemistry (IHC)

IHC staining was performed on a HCC tissue microarray (TMA). The expression levels were scored as proportion of immunopositive staining area (0%, 0; 1–25%, 1; 26–50%, 2; 51–75%, 3; 76–100%, 4) multiplied by intensity of staining (0, negative; 1, weak; 2, moderate; 3, intense). The scores were independently rendered by two pathologists (Dr. Yang YF and Dr. Zhang MF). The median IHC score (4.0) was chosen as the cut-off value to define high and low expression.

### Colony formation

Stable cells were constructed. Cells were collected and seeded in 6-well plates at a density of 1.0 × 10^3^ per well, and then incubated for 14 days. Colonies were fixed with methanol, stained with 0.1% crystal violet and counted.

### Transwell assay

A total of 3.0 × 10^4^ cells were re-suspended in 200 μl of serum-free medium and placed in the upper compartment of a Transwell chamber (Corning; 24-well insert, pore size: 8 mm). The lower chamber was filled with 15% fetal bovine serum as a chemo attractant and incubated for 48 h for the migration assay. After 48 h, the cells on the upper surface of the membrane were removed, and the cells on the lower surface were fixed and stained with 0.1% crystal violet. Five visual fields of each insert were randomly chosen and counted under a light microscope.

### Luciferase reporter assay

PLC8024 cells were co-transfected with miR-363-3p mimics or the negative control and psiCHECK-2-SPAG5–3’-UTR reporter. Cells were collected 36 h after transfection and analyzed with the Dual-Luciferase Reporter Assay System (Promega, CA, USA).

### Animal model

Male BALB/c nude mice aged 3–4 weeks were randomly divided into two groups. Four million cells were implanted subcutaneously under the right armpits into the flanks of the mice. Each group included 6 mice. Tumor size and body weight were measured once every 3 days. Four weeks later, the mice were sacrificed, and tumor weight and size were measured again. Volumes were calculated using the following formula: Volume (mm^3^) = [width^2^ (mm^2^) × length (mm)]/2. For metastasis observations, five-week-old male nude BALB/c mice were injected with 5 × 10^5^ cells via the tail vein. Six weeks later, the mice were killed. The lungs of the mice were fixed and stained with hematoxylin and eosin. Lung metastasis was quantified by counting the number of tumor nodule in 10 randomly selected high-power fields. All animal studies were conducted with the approval of the Medical Experimental Animal Care Commission of SYSUCC.

### Statistical analysis

The Student’s t-test was used for comparisons between groups. Kaplan–Meier analyses were used for survival analysis. Differences were considered significant for *P*-values less than 0.05. All data from three separate experiments are presented as mean ± SEM.

## Results

### SPAG5 expression is increased and associated with poor outcomes in HCC

**Fig. 1:**
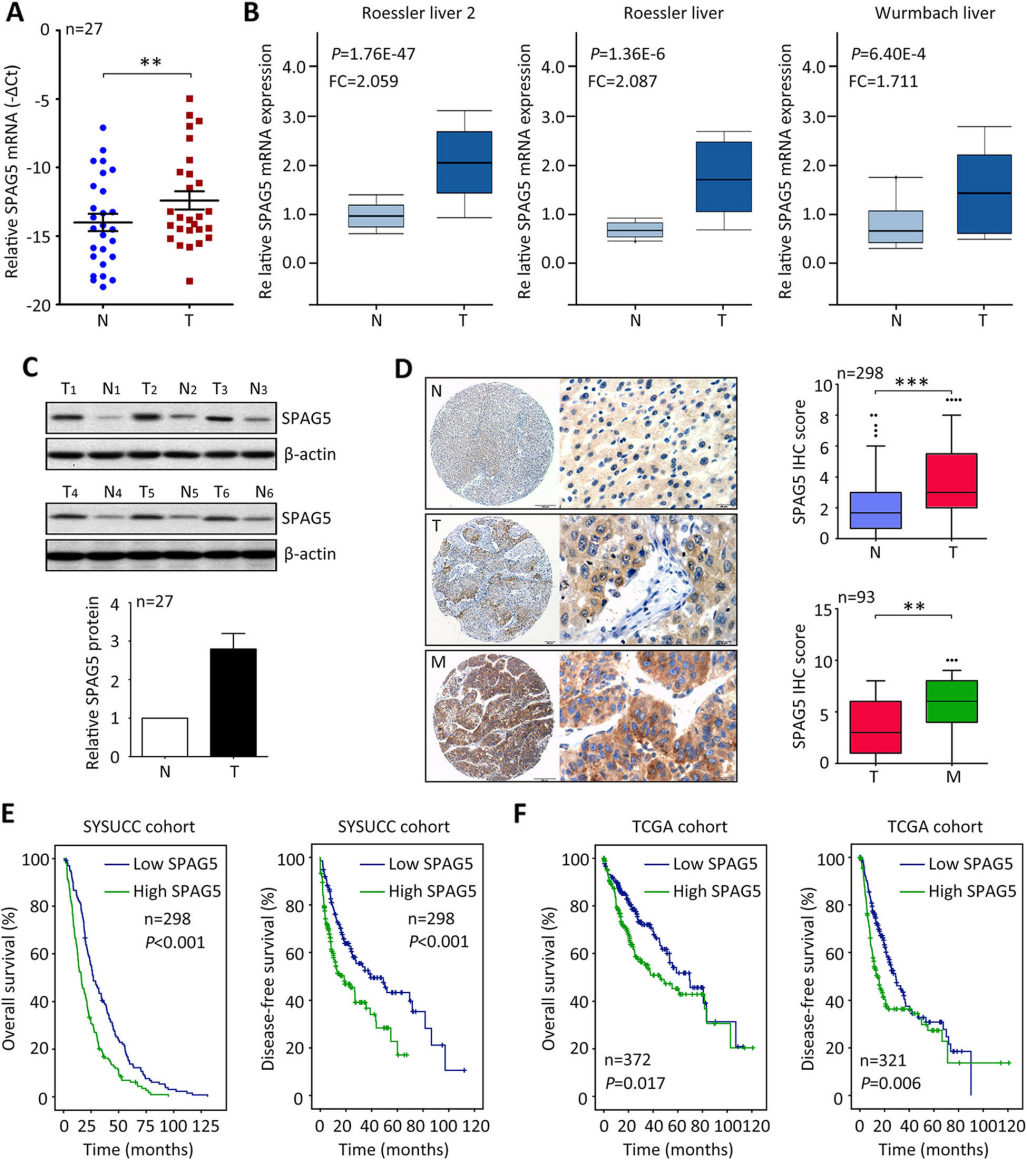
SPAG5 expression is increased in HCC and associated with poor outcomes. **a** The mRNA expression of SPAG5 in 27 pairs of HCC (T) and nontumorous (N) tissues was determined by qRT-PCR. **b** Studies from *Oncomine* datasets presented the increase of SPAG5 mRNA in HCC samples. **c** The protein expression of SPAG5 in HCC cases were examined by western blot. The representative images and the statistical analysis were indicated. **d** The expression of SPAG5 in nontumorous, HCC and metastatic tissues was detected by IHC. The representative images and the IHC scores were shown. **e** The clinical significance of SPAG5 expression in overall and disease-free survivals was evaluated in SYSUCC cohort by Kaplan-Meier survival analyses. **f.** The prognostic value of SPAG5 was confirmed in TCGA cohort. All **P < 0.01, ***P < 0.001

The expression of SPAG5 was firstly examined in fresh HCC tissues, using qRT-PCR, western blot and IHC. Results showed that no gene amplification was found for SPAG5 in HCC (Additional file 1: Figure S1). The mRNA level of SPAG5 in 27 paired HCC tissues was frequently up-regulated, compared to the corresponding nontumorous tissues (Fig. 1a). This was validated by other liver studies in Oncomine dataset (Fig. 1b). Consistently, the protein expression of SPAG5 in fresh HCC specimens was elevated by 2.65 folds on average (Fig. 1c). The protein expression of SPAG5 in HCC cell lines was much higher than that in immortalized liver cell line (L-02) (Additional file 2: Figure S2). Results of TMA-based IHC in SYSUCC cohort containing 298 patients with HCC demonstrated that more SPAG5 was expressed in HCC tissues (Fig. 1d). The patients were divided into two groups according to the median IHC score (4.0): high SPAG5 and low SPAG5. High expression of SPAG5 was associated with larger tumor size, poor tumor differentiation, advanced TNM stage, more lymph node metastasis and tumor vascular invasion (Table 1). Furthermore, a cohort consisting of 93 patients with tumor embolus was recruited to determine the expression of SPAG5 in metastatic tumor tissues. Increased expression of SPAG5 was found in tumor metastasis, compared with the primary tumor (Fig. 1d).

**Table 1:** Correlation of SPAG5 expression and clinical features of HCC patients

Variable	SPAG5			
	All cases	Low expression	High expression	*P* value^a^
Age (years) ^b^				0.524
< 49	143	68 (47.6%)	75 (52.4%)	
≥ 49	155	68 (43.9%)	87 (56.1%)	
Gender				
Male	272	125 (46.0%)	147 (54.0%)	0.721
Female	26	11 (42.3%)	15 (57.7%)	
HBV				0.858
Positive	238	108 (45.4%)	130 (54.6%)	
Negative	60	28 (46.7%)	32 (53.3%)	
AFP (ng/ml)				0.933
< 20	76	35 (46.1%)	41 (53.9%)	
≥ 20	222	101 (45.5%)	121 (54.5%)	
Cirrhosis				0.101
Yes	264	116 (43.9%)	148 (56.1%)	
No	34	20 (58.8%)	14 (41.2%)	
Tumor size (cm)				0.039
< 5	67	38 (56.7%)	29 (43.3%)	
≥ 5	231	98 (42.4%)	133 (57.6%)	
Tumor multiplicity				0.058
Single	182	91 (50.0%)	91 (50.0%)	
Multiple	116	45 (38.8%)	71 (61.2%)	
Differentiation				0.034
Well-Moderate	219	108 (49.3%)	111 (50.7%)	
Poor-undifferentiated	79	28 (35.4%)	51 (64.6%)	
Vascular invasion				0.008
Yes	62	19 (30.6%)	43 (69.4%)	
No	236	117 (49.6%)	119 (50.4%)	
LNM				0.034
Yes	24	6 (25.0%)	18 (75.0%)	
No	274	130 (47.4%)	144 (52.6%)	
Tumor capsule				0.391
Absent	181	79 (43.6%)	102 (56.4%)	
Present	117	57 (48.7%)	60 (51.3%)	
TNM				0.045
I-II	152	78 (51.3%)	74 (48.7%)	
III-IV	146	58 (39.7%)	88 (60.3%)	

^a^ Chi-square test, ^b^Median age, *AFP* alpha-fetoprotein, *HBsAg* hepatitis B surface antigen, *LNM* lymph node metastasis

The prognostic implication of SPAG5 in HCC was next explored. In SYSUCC cohort, patients with high SPAG5 expression were likely to survive shorter and experience tumor relapse in a shorter time, compared with those with low SPAG5 expression (Fig. 1e). The median overall survivals in high SPAG5 and low SPAG5 groups were 16.0 and 27.1 months, respectively. Multivariate analyses using Cox regression model revealed SPAG5 as an independent prognostic factor for overall survival in HCC (Table 2). The data from TCGA database confirmed the prognostic value of SPAG5 in HCC (Fig. 1f). The 5-year survival in high SPAG5 and low SPAG5 groups were 41% and 52% respectively. These data suggest that overexpression of SPAG5 serves as a promising factor for the prognosis of patients with HCC.

**Table 2:** Univariate and multivariate analyses of SPAG5 expression and overall survival

Variables	Univariate analysis		Multivariate analysis	
	HR (95% CI)	*P* value	HR (95% CI)	*P* value
Overall survival				
Age (< 49 vs. ≥49 years)	0.954 (0.757–1.203)	0.692		
Gender (female vs. male)	1.008 (0.667–1.522)	0.971		
HBV (positive vs. negative)	1.094 (0.816–1.466)	0.549		
Liver cirrhosis (yes vs. no)	0.877 (0.612–1.256)	0.474		
Tumor size (< 5 vs. ≥5 cm)	1.560 (1.176–2.069)	0.002	1.217 (0.877–1.689)	0.240
Tumor multiplicity (single vs. multiple)	1.361 (1.072–1.729)	0.011	0.890 (0.660–1.202)	0.448
Tumor capsule (absent vs. present)	0.528 (0.411–0.677)	0.000	0.645 (0.490–0.850)	0.002
AFP (< 20 vs. ≥20 ng/mL)	1.526 (1.167–1.994)	0.002	1.329 (1.000–1.766)	0.050
Vascular invasion (yes vs. no)	2.081 (1.556–2.784)	0.000	1.243 (0.894–1.729)	0.196
Tumor differentiation	1.363 (1.045–1.777)	0.022	1.116 (0.846–1.473)	0.438
LNM (yes vs. no)	1.652 (1.076–2.538)	0.022	1.290 (0.824–2.019)	0.266
TNM (I-II vs. III-IV)	1.905 (1.498–2.424)	0.000	1.356 (0.959–1.917)	0.085
SPAG5 expression (low vs. high)	0.954 (0.757–1.203)	0.000	1.697 (1.322–2.178)	0.000

*AFP* a-fetoprotein, *HBsAg* hepatitis B surface antigen, *LNM* lymph node metastasis, *HR* hazard ratio, *CI* confidence interval

### SPAG5 promotes cell proliferation in HCC

**Fig. 2:**
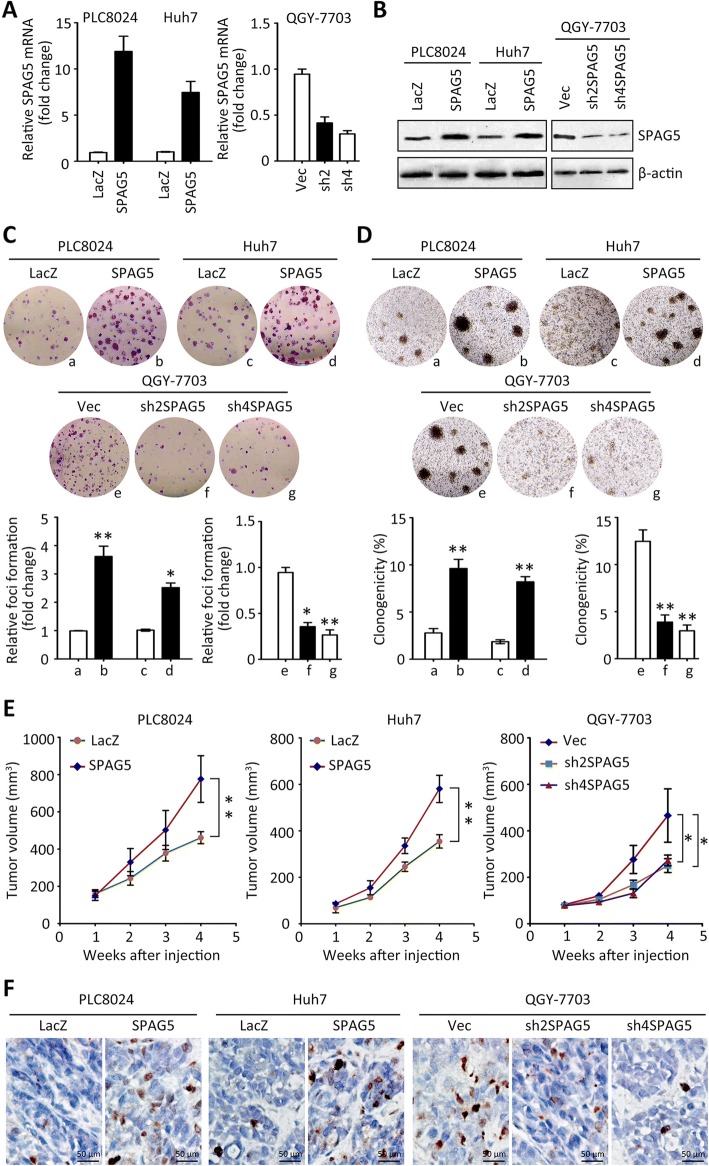
SPAG5 promotes cell growth in HCC. **a** PLC8024 and Huh7 cells were transfected with SPAG5 overexpression vectors. QGY-7703 cells were incubated with SPAG5 shRNAs. Stable cell lines were established by G418 screening. The mRNA levels of SPAG5 in were determined by qRT-PCR. **b** The protein expression of SPAG5 in the stable cell lines was examined by western blot. **c** The effect of SPAG5 on cell proliferation was determined by foci formation assays. The representative images and the fold change of foci formation were shown. **d** Colony formation in soft agar was performed to validate the impact of SPAG5 on cell growth. **e** Cells with SPAG5 overexpression or knockdown were injected into the right flank of null mice for 4 weeks. The tumor volumes were measured every week and indicated by curves. **f** Tumors were sectioned at day 28. Ki67 staining was conducted to demonstrate the cell proliferation. All *P < 0.05, **P < 0.01

The biological function of SPAG5 in HCC was next investigated. PLC8024 and Huh7 cells were stably transfected with SPAG5 overexpression vector, and QGY-7703 cells were treated with SPAG5 shRNAs. The mRNA and protein expression of SPAG5 in stable cell lines were examined by qRT-PCR and western blot (Fig.2a and b). In HCC cells, overexpression of SPAG5 markedly enhanced the cell proliferation. Foci formation and soft agar assays demonstrated that SPAG5 significantly increased the frequency of colony formation on solid plates (Fig. 2c) and in soft agar (Fig. 2d). In contrast, the silence of SPAG5 resulted in reduced colonies in QGY-7703 cells. These findings indicated that SPAG5 promoted cell growth in both anchorage-dependent and -independent manners.

In vivo tumor formation assay was used to examine the effect of SPAG5 on tumor growth. Tumors were found in 6/6 and 4/6 of mice injected with SPAG5-transfected and empty vector-transfected PLC8024 cells, respectively. The related numbers in Huh7 cells were 6/6 and 5/6. Tumors formed by PLC8024 and Huh7 cells with SPAG5 overexpression grew much faster than those in control groups. However, the tumors derived from QGY-7703 cells transfected with SPAG5 shRNAs were much smaller and lighter than those in control group. Tumor formation was observed only in 3/6 of mice injected with SPAG5 shRNAs-transfected QGY-7703 cells, but in 5/6 of mice in control group (Fig. 2e). The cell proliferation in tumors was evaluated by Ki67 staining. Results showed that cell proliferation was enhanced by SPAG5 overexpression but attenuated by SPAG5 knockdown (Fig. 2f).

### SPAG5 facilitates cell migration in HCC

**Fig. 3:**
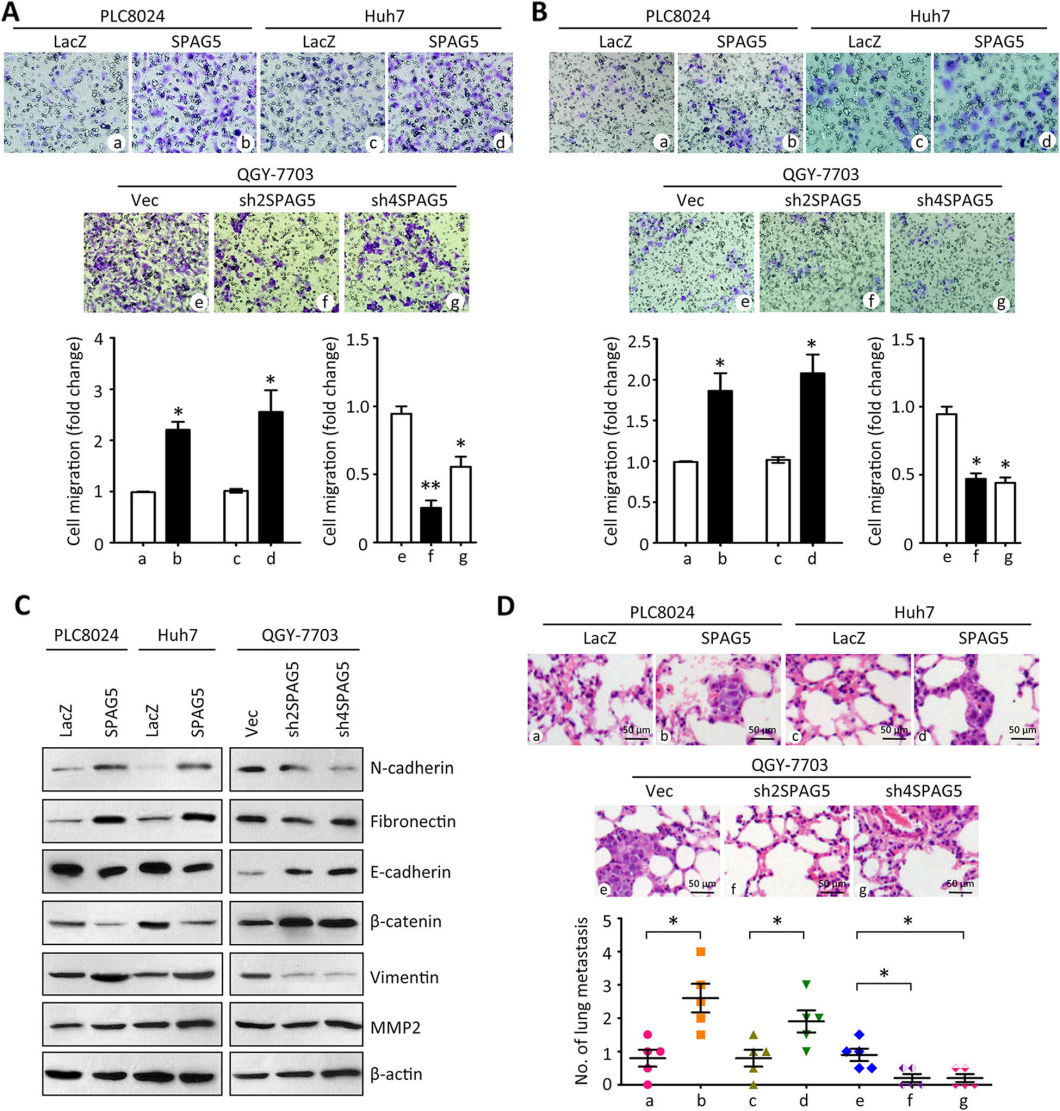
SPAG5 facilitates cell migration in HCC. **a** Cells with SPAG5 overexpression or silence were subjected to Transwell assays. The migrated cells were stained with 0.05% crystal violet and counted. The representative images and the fold change of cell migration were presented. **b** Invasion assays were performed to determine the effect of SPAG5 on cell invasion. **c** Proteins extracted from stable cell lines were subjected to western blot to detect the expressions of EMT-related markers, such as N-cadherin and MMP2. **d** Stable cells were injected into the mice via the tail vein. At day 40, the lungs were dissected. HE staining was performed to indicate the tumor metastasis in the lungs. Representative images and the statistical analyses were shown. All *P < 0.05, **P < 0.01

Since clinical data revealed that SPAG5 expression was correlated with metastatic features in HCC, the effect of SPAG5 on HCC cell migration was next determined. Transwell assays were performed to show that SPAG5 overexpression increased the migrated cells in PLC8024 and Huh7 cells, whereas SPAG5 depletion inhibited the cell movement in QGY-7703 cells (Fig. 3A). Invasion assays demonstrated that SPAG5 enhanced the ability of cell invasion in HCC cells (Fig. 3b). These data indicated SPAG5 might be involved in tumor metastasis. Western blot showed that the expression of Fibronectin, Vimentin, N-cadherin and MMP2 was induced by SPAG5, but reduced by SPAG5 knockdown in HCC cells. In contrast, the expression of E-cadherin and β-catenin was down-regulated by SPAG5 overexpression (Fig. 3c). In vivo tumor metastasis models demonstrated that more lung nodules were depicted in mice injected with PLC8024 and Huh7 cells with SPAG5 overexpression. shRNAs against SPAG5 significantly reduced the tumor metastasis in mice (Fig. 3d). These data suggested SPAG5 triggered the epithelial-mesenchymal transition (EMT) process in HCC cells to facilitate cell migration.

### SPAG5 is targeted by miR-363-3p in HCC

**Fig. 4:**
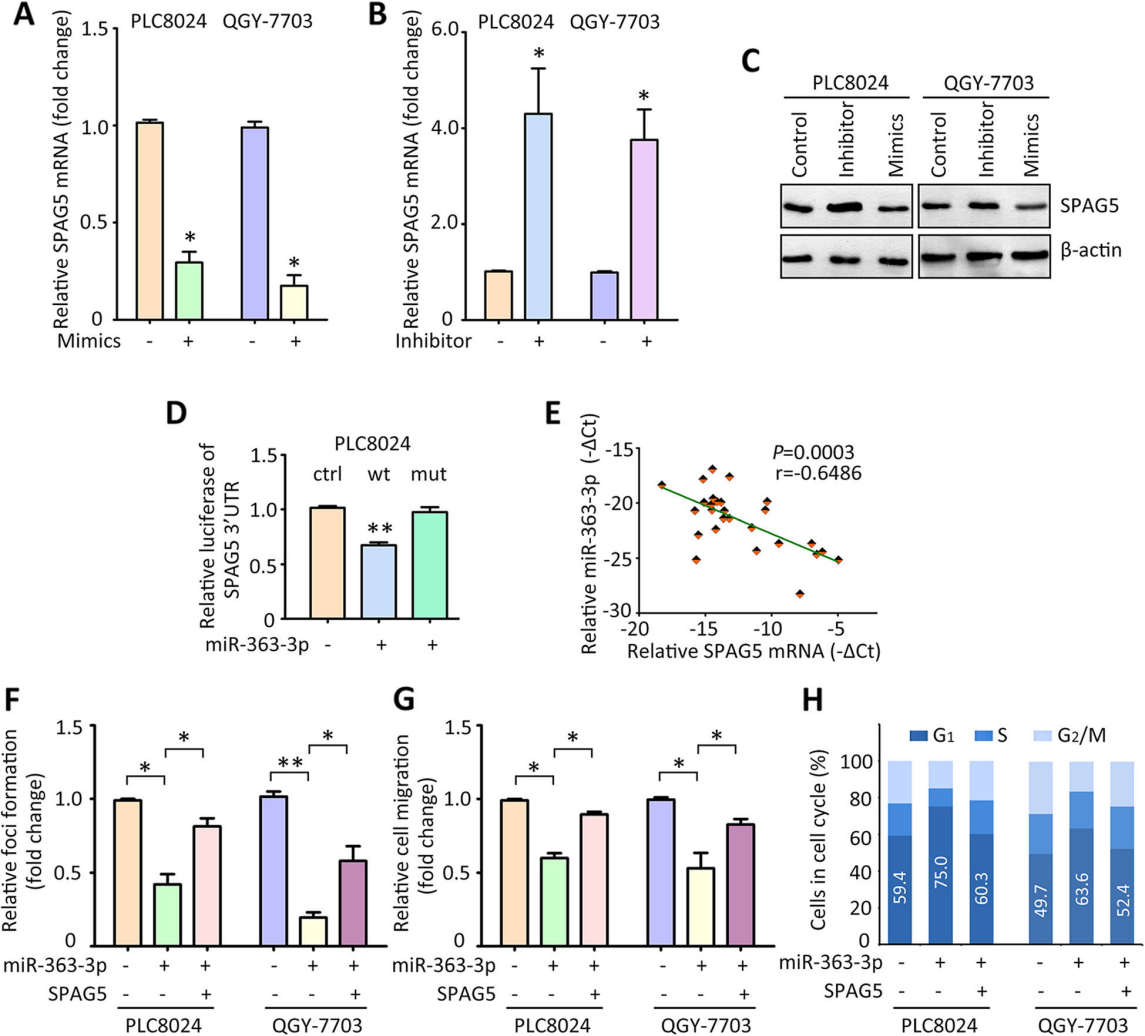
SPAG5 is targeted by miR-363-3p. **a** PLC8024 and QGY-7703 cells were transfected with miR-363-3p mimics for 36 h. The SPAG5 mRNA levels were determined by qRT-PCR. **b** Cells were treated with miR-363-3p inhibitor for 36 h. The SPAG5 mRNA levels were determined by qRT-PCR. **c** Proteins from cells treated with miR-363-3p mimics or inhibitors were subjected to western blot to examine the alteration of SPAG5 protein. **d** Luciferase reporter assays were performed to indicate the effect of miR-363-3p on the activity of SPAG5 mRNA 3’UTR. **e** The correlation of SPAG5 mRNA and miR-363-3p was determined in 27 HCC samples. **f** Cells expressing miR-363-3p were transfected with SPAG5 overexpression vector for 24 h. The cell proliferation was examined by foci formation assays. **g** The impact of SPAG5 in miR-363-3p-mediated cell migration was determined by Transwell assays. **h** The cell cycle analyses were performed in cells with miR-363-3p and SPAG5. The percentage of cells in G1 phase was indicated. *P < 0.05, **P < 0.01

The mechanism responsible for SPAG5 upregulation in HCC was next explored. Seven microRNAs were predicted by two bioinformatic algorithms (Targetscan and miRanda) to be the potential upstream regulator of SPAG5, including two tumor suppressors (miR-539-5p and miR-363-3p) (Additional file 3: Figure S3A). Since miR-539-5p has been reported to modulate the expression of SPAG5 in HCC cells, miR-363-3p, which was down-regulated in HCC cells (Additional file 4: Figure S4), was chosen for further studies. A putative site for the binding of miR-363-3p and SPAG5 3’UTR was identified (Additional file 3: Figure S3B). Re-introduction of miR-363-3p into PLC8024 and QGY-7703 resulted in a significant decrease of SPAG5 mRNA (Fig. 4a), whereas suppression of miR-363-3p by its specific inhibitor led to the increase of SPAG5 mRNA (Fig. 4b). Consistently, the protein level of SPAG5 was downregulated by miR-363-3p mimics but upregulated by its inhibitor in both HCC cell lines (Fig. 4c). Dual luciferase reporter assays showed that overexpression of miR-363-3p markedly inhibited the activity of wild type SPAG5 3’UTR but not the mutant 3’UTR (Fig. 4d). In clinical samples, the expression of miR-363-3p was reversely associated with SPAG5 mRNA (Fig. 4e). These data suggest that miR-363-3p is capable of modulating the expression of SPAG5 in HCC cells.

Previous literatures reported that miR-363-3p functioned as a tumor suppressor in HCC, which was validated by our studies (Fig. 4f-h). The effect of SPAG5 on miR-363-3p-mediated phenotypes was determined. Overexpression of SPAG5 increased the colony formation and cell migration in HCC cells expressing miR-363-3p (Fig. 4F and G). Furthermore, the G1 phase arrest caused by miR-363-3p was relieved by the ectopic expression of SPAG5 (Fig. 4h). Collectively, these data suggest that SPAG5 contributes to the tumor suppressive activity of miR-363-3p.

### SPAG5 interacts with CEP55 in HCC

**Fig. 5:**
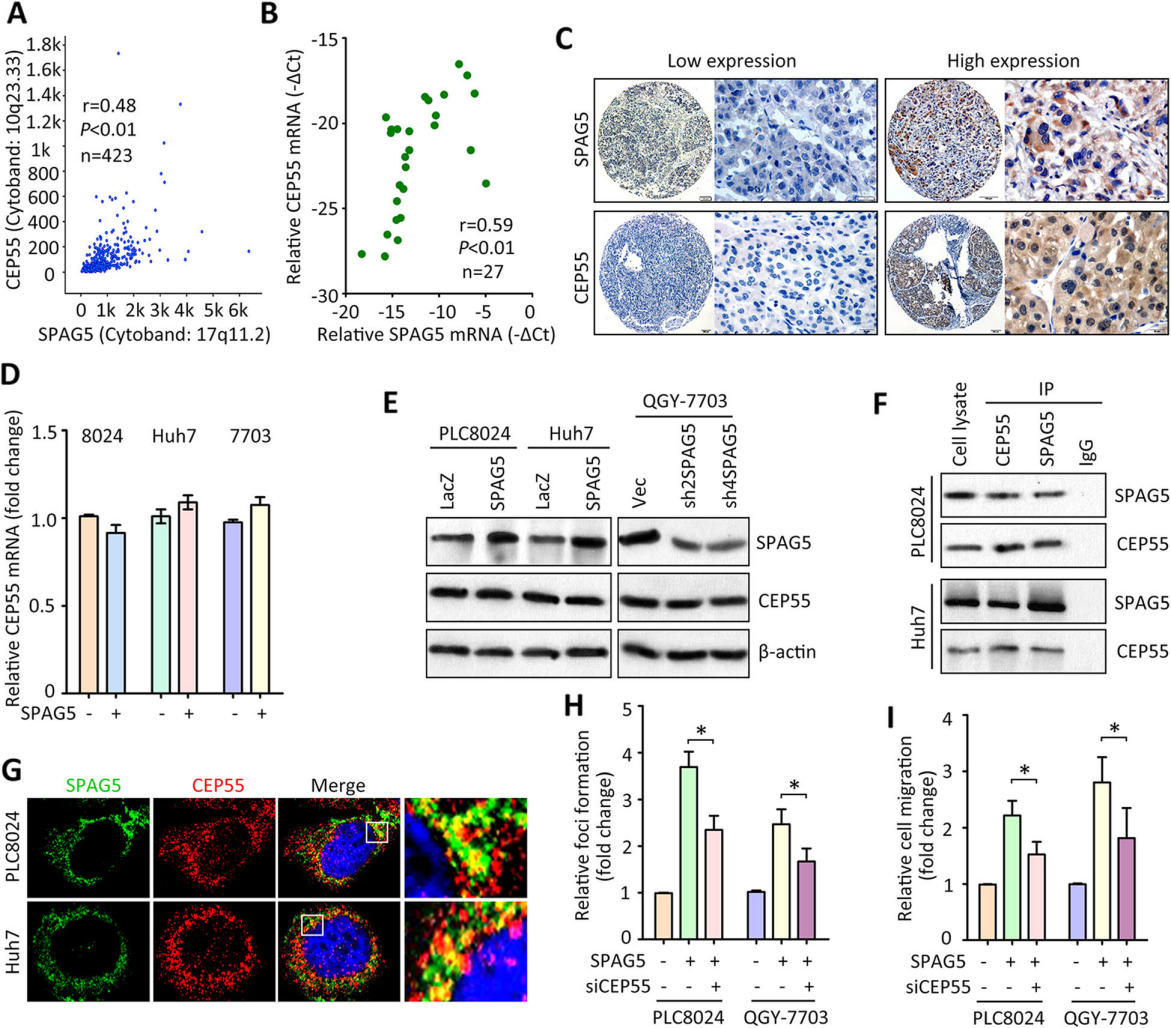
SPAG5 interacts with CEP55. **a** A positive correlation between SPAG5 mRNA and CEP55 mRNA was found in TCGA cohort. **b** The correlation of SPAG5 mRNA and CEP55 mRNA in fresh HCC tissues was identified. **c** Patients with high expression of SPAG5 protein in SYSUCC cohort were frequently accompanied with high expression of CEP55 protein. **d** Cells were transfected with SPAG5 overexpression vector. The mRNA level of CEP55 was determined. **e** The protein expression of CEP55 was examined in cells with SPAG5 overexpression or knockdown. **f** Proteins extracted from HCC cells were incubated with antibody for CEP55 or SPAG5. After deposited by Protein A/G-agarose, samples were subjected to western blot to investigate the interaction. **g** Cells were fixed by 4% PFA and incubated with antibodies overnight at 4 °C. After stained by fluorescence secondary antibodies and DAPI, cells were observed under confocal fluorescence microscope. **h** Cells expressing SPAG5 were transfected with CEP55 siRNA for 24 h. The cell proliferation was examined by foci formation assays. **i** The impact of CEP55 in SPAG5-mediated cell migration was determined by Transwell assays. *P < 0.05

The underlying mechanism via which SPAG5 exerted oncogenic functions towards HCC was investigated. According to the expression of SPAG5 mRNA in TCGA datasets, patients were divided into two groups. Genes co-expressing with SPAG5 was determined and shown by heatmap (Additional file 5: Figure S5A and Additional file 6: Table S1). Gene Set Enrichment Analysis (GSEA) indicated that pathway involved in akapcentrosome was activated in cases with high SPAG5 expression (Additional file 5: Figure S5B). SPAG5 expression was positively correlated with the expression of CEP55 that contributes to the centrosome modulation, in both TCGA and SYSUCC samples (Fig. 5a-b). HCC patients with high SPAG5 were frequently accompanied with high expression of CEP55 (Fig. 5c). In HCC cells, ectopic SPAG5 expression did not alter the expression of CEP55 at both mRNA and protein levels (Fig. 5d-e), which implies that SPAG5 can not modulate the expression of CEP55. Instead, CEP55 was detectable in the precipitant mediated by the specific antibody of SPAG5 in both PLC8024 and Huh7 cells (Fig. 5f). Confocal assays demonstrated the co-localization of SPAG5 and CEP55 in the cytoplasm of the two cell lines (Fig. 5g). Functionally, the knockdown of CEP55 significantly attenuated SPAG5-promoted cell proliferation and migration (Fig. 5h and i). These data indicate that SPAG5 exhibits pro-HCC activities through interacting with CEP55.

### SPAG5 triggers the PI3K/AKT signaling pathway in HCC

**Fig. 6:**
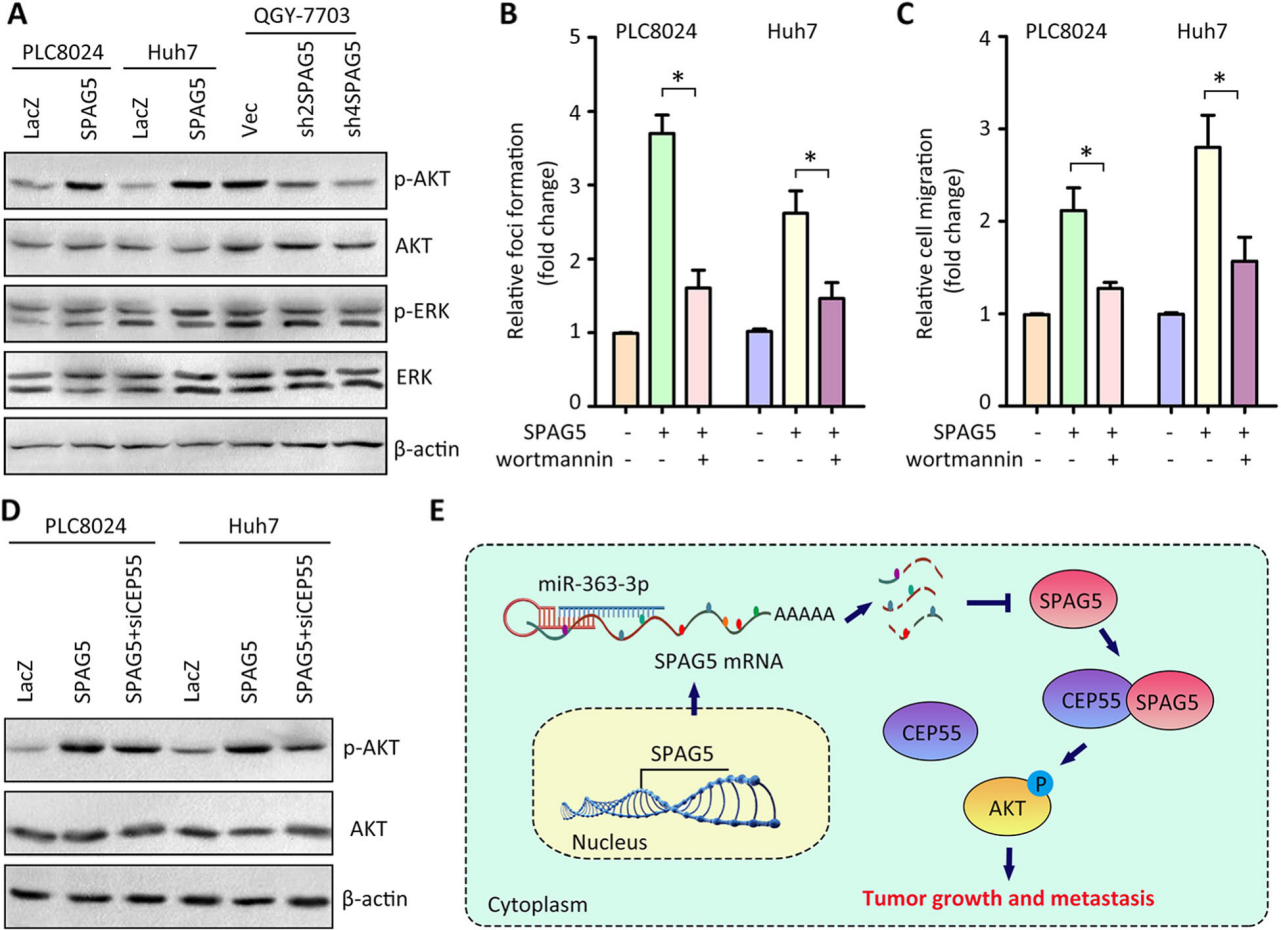
SPAG5 triggers PI3K/AKT pathway. **a** Proteins from stable cell lines were subjected to western blot to examine the expression of phosphorylated AKT (p-AKT), AKT, phosphorylated ERK1/2 (p-ERK1/2) and ERK1/2. **b** Cells with SPAG5 overexpression were incubated with wortmanin (a specific inhibitor of PI3K) for 24 h. The cell proliferation was examined by foci formation assays. **c** The impact of activation of PI3K/AKT pathway in SPAG5-mediated cell migration was determined by Transwell assays. **d** Cells expressing SPAG5 were transfected with CEP55 siRNA for 24 h. The expression of p-AKT was examined by western blot. **e** A schematic diagram of SPAG5-promted cell growth and metastasis was shown. All *P < 0.05

Current studies have shown that CEP55 promotes tumor progression via PI3K/AKT/mTOR pathway. The effect of SPAG5 on the activation of PI3K/AKT signaling was next determined. Western blot showed that the phosphorylation of AKT at Ser473, but not of ERK1/2 at Thr202/Tyr204, was increased in HCC cells with SPAG5 overexpression, and decreased in cells with SPAG5 silence (Fig. 6a). Upon the treatment of wortmannin (an inhibitor for PI3K/AKT pathway), the SPAG5-mediated cell growth and migration in HCC cells were dramatically suppressed (Fig. 6b and c). Furthermore, the activation of AKT by SPAG5 was partly blocked by the incubation of CEP55 siRNA (Fig. 6d). These data suggest that SPAG5 triggers the PI3K/AKT pathway in HCC via the interaction with CEP55.

## Discussion

HCC represents the fifth most common malignant tumor worldwide and has been becoming a global threat to human life [1]. Although HCC has been extensively studied, the detailed molecular events in the disease’s development are still elusive. More potential markers useful for the prediction of HCC progression and prognosis are required to provide clinical significances. In this study, we showed that high SPAG5 expression was associated with aggressiveness and poor prognosis of HCC patients independent of other clinical features. Overexpression of SPAG5 significantly enhanced the proliferative and migrated ability of HCC cells via the interaction with CEP55 to trigger the PI3K/AKT signaling pathway (Fig. 6e). Our data suggest SPAG5 functions as an oncogene to promote HCC and therefore serves as a promising therapeutic target for the intervention of HCC.

Identification of proteins with prognostic value may contribute to tumor classification and the development of novel therapies against human cancers. The clinical impact of SPAG5 has been well documented in breast cancer. Copy number aberration of SPAG5, as well as the high expression of SPAG5 transcript and protein, contributed to the worse overall and cancer-specific survival of patients with breast cancer [15]. Upregulation of SPAG5 in cervical cancer was reported to associate with poor prognosis [12]. In two independent cohorts containing 670 HCC patients, we found that patients with increased expression of SPAG5 were frequently accompanied with shorter survival. This might be attributed to the fact that high expression of SPAG5 was correlated with unfavorable clinical parameters, including poor tumor differentiation, larger tumor size, advanced TNM stage, tumor vascular invasion and lymph node metastasis. Furthermore, our in vitro and in vivo data demonstrated that cancer cells with SPAG5 overexpression were more aggressive. These data suggest SPAG5 is involved in the progression of human cancers.

Post-transcriptional modulation of mRNA is responsible for the dysregulation of microtubule proteins. SPAG5 was previously demonstrated to be targeted by miR-539 in prostate cancer [16]. In this study, we identified miR-363-3p as an upstream regulator to suppress the expression of SPAG5 in HCC cells. Overexpression of miR-363-3p markedly inhibited the cell growth and migration, which could be markedly attenuated by SPAG5 overexpression. Further data indicated that miR-363-3p reduced SPAG5 expression via suppression of the promoter activity of SPAG5 mRNA. The role of miR-363-3p in HCC was reported by Zhou and colleagues. Their data showed that miR-363-3p blocked the cell proliferation via S1PR1-mediated silence of ERK and STAT3 signaling pathways [17].

The investigation of the biological function of SPAG5 in HCC demonstrated that SPAG5 exerted oncogenic activities to promote tumor growth and metastasis via interaction with CEP55. CEP55 (FLJ10540, C10orf3) was mapped to the 10q23 chromosomal region, with a cellular localization to centrosome throughout mitosis [18]. Overexpression of CEP55 has been reported in colon cancer [19], head and neck cancer [20], lung cancer [21], oral cavity squamous cell carcinoma [22] and HCC [23]. High expression of CEP55 activated prosurvival signaling pathways, resulting in cancer cell proliferation and migration. Chen et al. showed that CEP55 facilitated cellular transformation through the activation of PI3K/AKT pathway via protein interaction [24]. In the present study, SPAG5 physically bound to CEP55. The inhibition of PI3K/AKT signaling remarkably abolished the SPAG5-promoted cell growth. Collectively, these data suggest SPAG5/CEP55/AKT axis might be a potential therapeutic target in HCC.

## Conclusions

In summary, we show that increased expression of SPAG5 in HCC was closely correlated with poor outcomes, indicating that SPAG5 serves a promising prognostic factor in HCC. Our data demonstrate SPAG5 functions as an oncogene via CEP55-mediated PI3K/AKT pathway. The newly identified miR-363-3p/SPAG5/CEP55 axis may represent a potential therapeutic target for the clinical intervention of HCC.

## Additional files

Additional file 1: **Figure S1.** The determination of gene amplification of SPAG5 in HCC. **A.** TCGA data showed 1.1% of HCC cases were accompanied with gene amplification. **B.** Negative signal for gene amplification determined by FISH using SPAG5 probe was depicted in HCC cases (Red, SPAG5; Green, chromosome 17). Lymphocytes were used as negative control, and breast cancer cells were used as positive control. (PDF 583 kb)

Additional file 2: **Figure S2.** The expression of SPAG5 in HCC cell lines were determined by western blot. (JPG 270 kb)

Additional file 3: **Figure S3.** The upstream microRNA for SPAG5 was predicted by two bioinformatic algorithms (Targetscan and miRanda). **A.** microRNAs targeting SPAG5 were predicted by Targetscan and miRanda. Seven microRNAs including miR-363-3p were overlapped. **B.** A putative binding site for miR-363-3p and SPAG5 was shown. Vector containing wild type or mutant 3’UTR of SPAG5 was constructed according to the sequence. (JPG 349 kb)

Additional file 4: **Figure S4.** The expression of miR-363-3p was examined in HCC cell lines by qRT-PCR. (JPG 213 kb)

Additional file 5: **Figure S5.** The GSEA analysis of SPAG5 in TCGA data. **A.** Patients in TCGA dataset were separated into two groups according to the expression of SPAG5. The heatmap showed the top 50 features for each phenotype. **B.** Gene Set Enrichment Analysis (GSEA) indicated that pathway involved in akapcentrosome was activated in cases with high SPAG5 expression. (JPG 1093 kb)

Additional file 6: **Table S1.** The top 50 genes co-expressing with SPAG5 in HCC samples were shown. (XLSX 14 kb)

## Abbreviations

EMT: Epithelial-mesenchymal transition
ERK: extracellular regulated protein kinases
HCC: hepatocellular carcinoma
PI3K: Phosphoinositide 3 kinase
SPAG5: Sperm-associated antigen 5
