# Supplementary figures and images for: SPAG5 interacts with CEP55 and exerts oncogenic activities via PI3K/AKT pathway in hepatocellular carcinoma
Source: Mol Cancer. 2018 Aug 8;17:117. doi: 10.1186/s12943-018-0872-3 (PMC6081940; doi:10.1186/s12943-018-0872-3)

**A**

1.1%

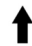

98.9%

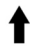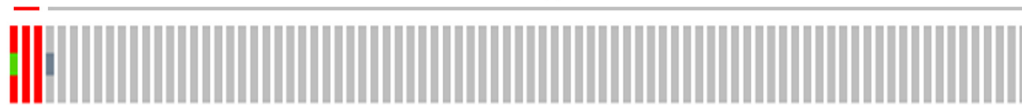

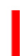 Amplification    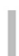 No alterations

**B**

Lymphocytes

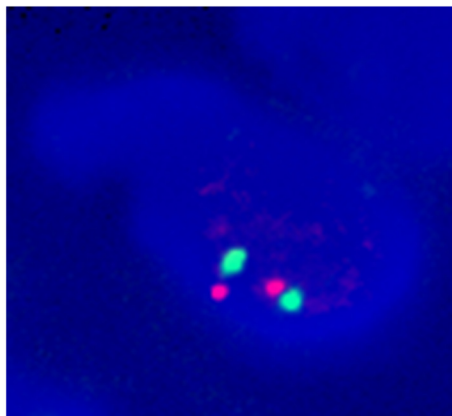

HCC Case 546895

Breast cancer cells

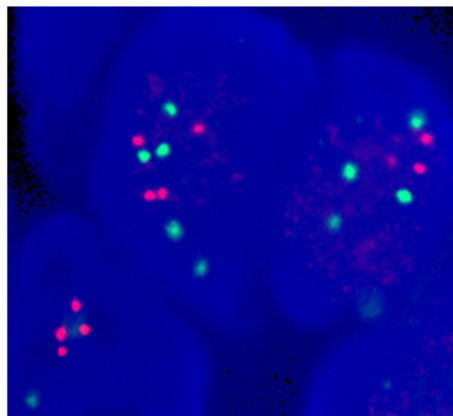

HCC Case 612548

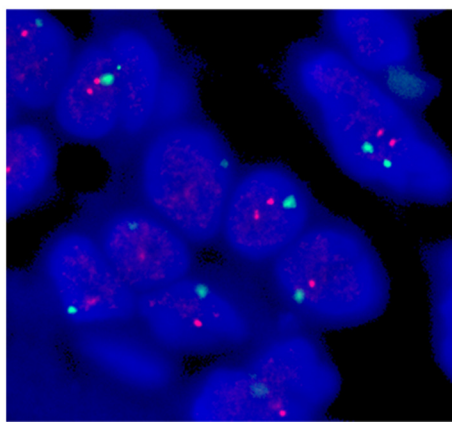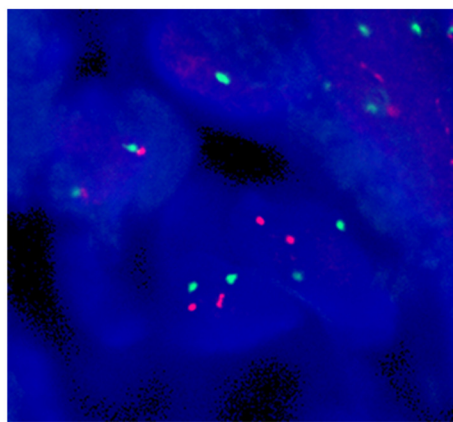

Supplement: Supplementary file 1 — Figure S1. The determination of gene amplification of SPAG5 in HCC. A. TCGA data showed 1.1% of HCC cases were accompanied with gene amplification. B. Negative signal for gene amplification determined by FISH using SPAG5 probe was depicted in HCC cases (Red, SPAG5; Green, chromosome 17). Lymphocytes were used as negative control, and breast cancer cells were used as positive control. (PDF 583 kb) [file 12943_2018_872_MOESM1_ESM.pdf]

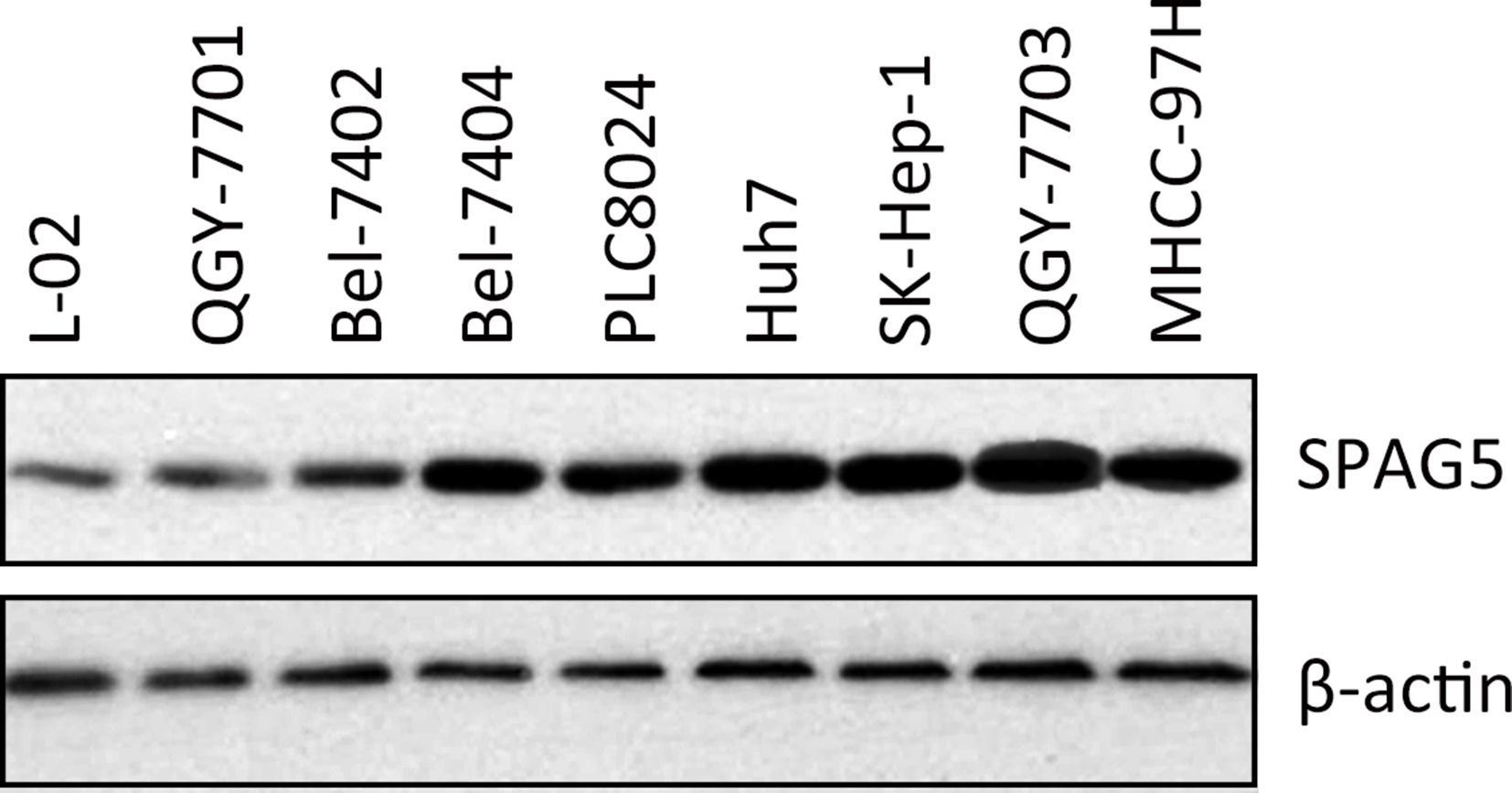

Supplement: Supplementary file 2 — Figure S2. The expression of SPAG5 in HCC cell lines were determined by western blot. (JPG 270 kb) [file 12943_2018_872_MOESM2_ESM.jpg]

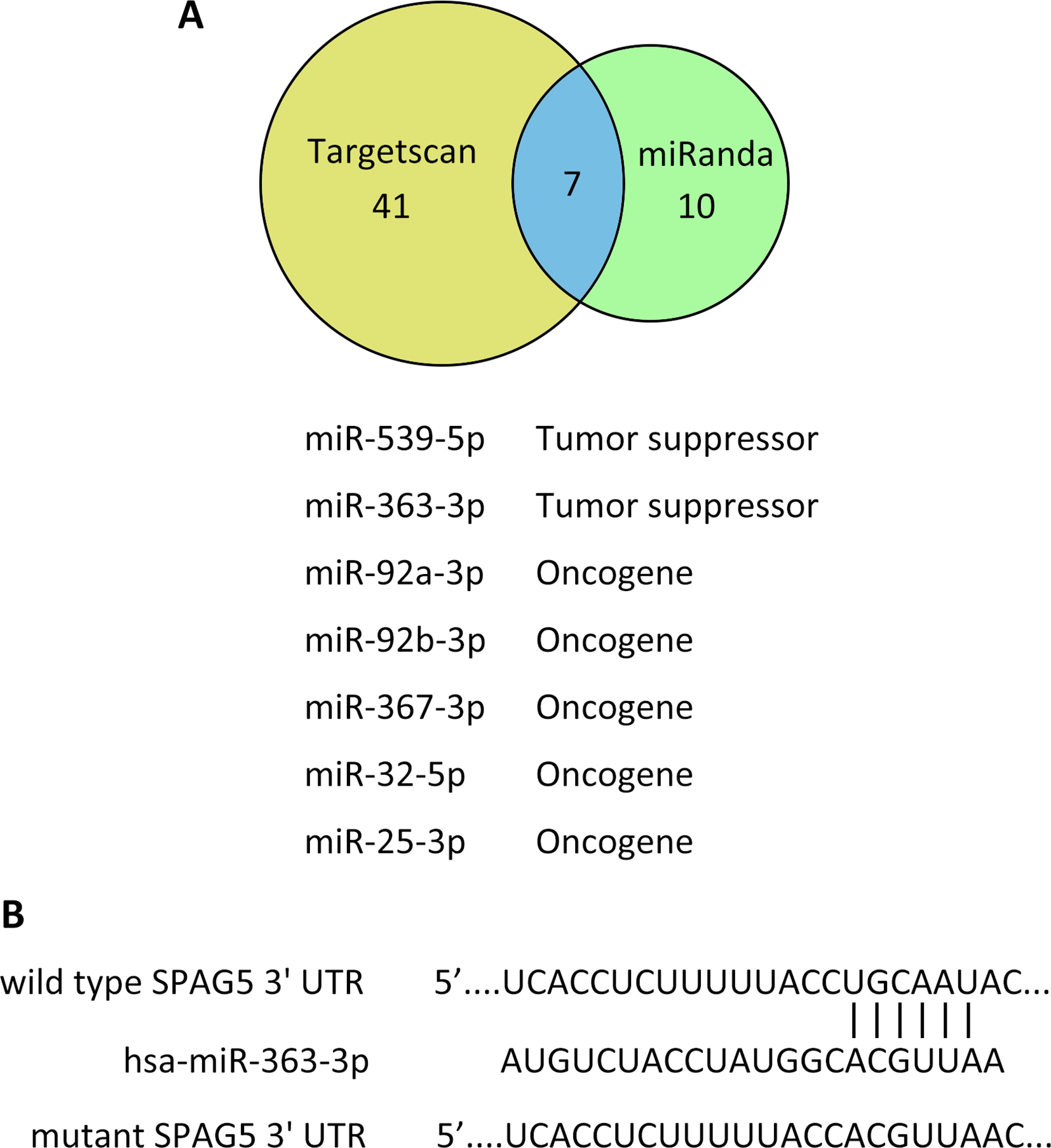

Supplement: Supplementary file 3 — Figure S3. The upstream microRNA for SPAG5 was predicted by two bioinformatic algorithms (Targetscan and miRanda). A. microRNAs targeting SPAG5 were predicted by Targetscan and miRanda. Seven microRNAs including miR-363-3p were overlapped. B. A putative binding site for miR-363-3p and SPAG5 was shown. Vector containing wild type or mutant 3’UTR of SPAG5 was constructed according to the sequence. (JPG 349 kb) [file 12943_2018_872_MOESM3_ESM.jpg]

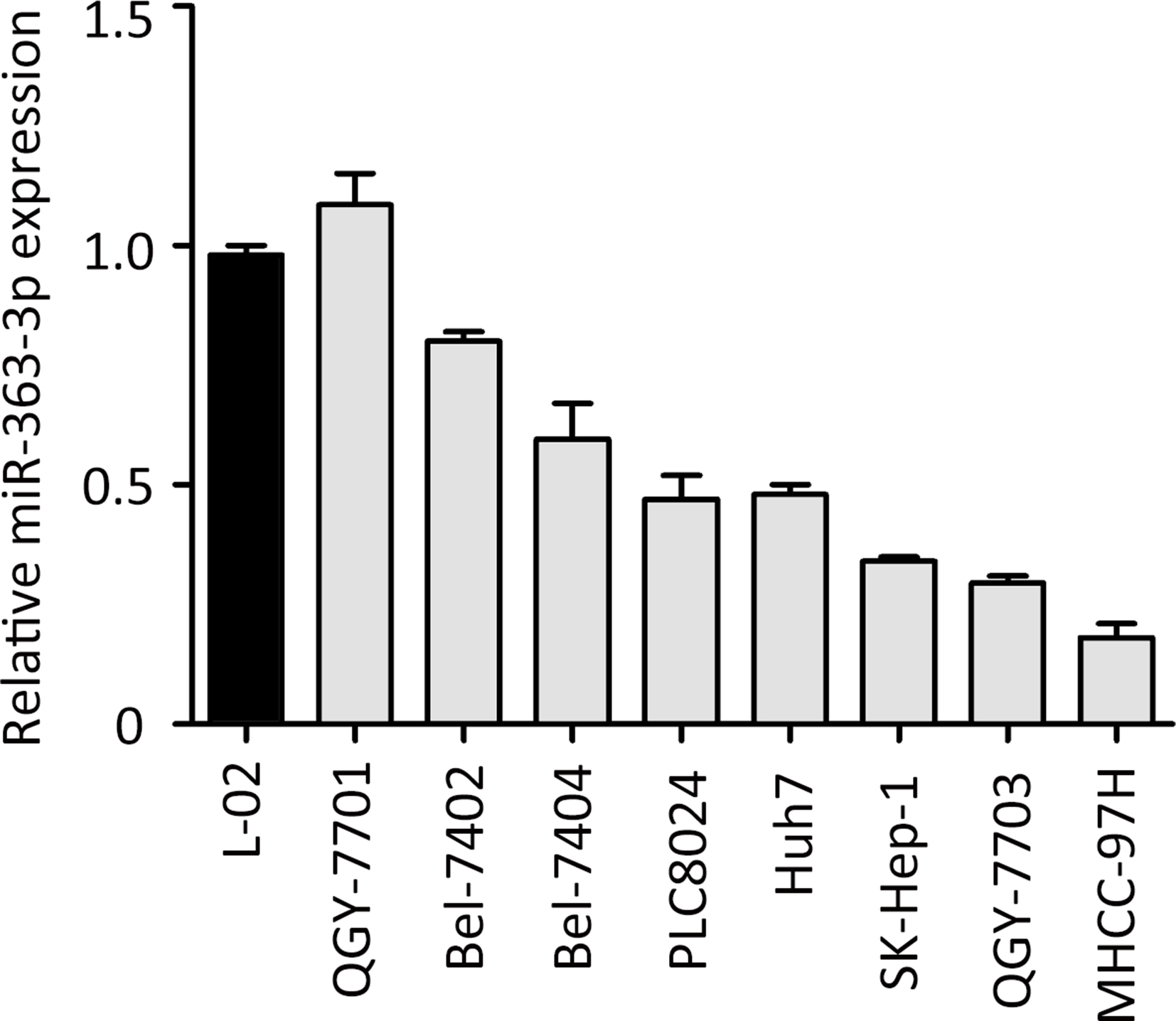

Supplement: Supplementary file 4 — Figure S4. The expression of miR-363-3p was examined in HCC cell lines by qRT-PCR. (JPG 213 kb) [file 12943_2018_872_MOESM4_ESM.jpg]

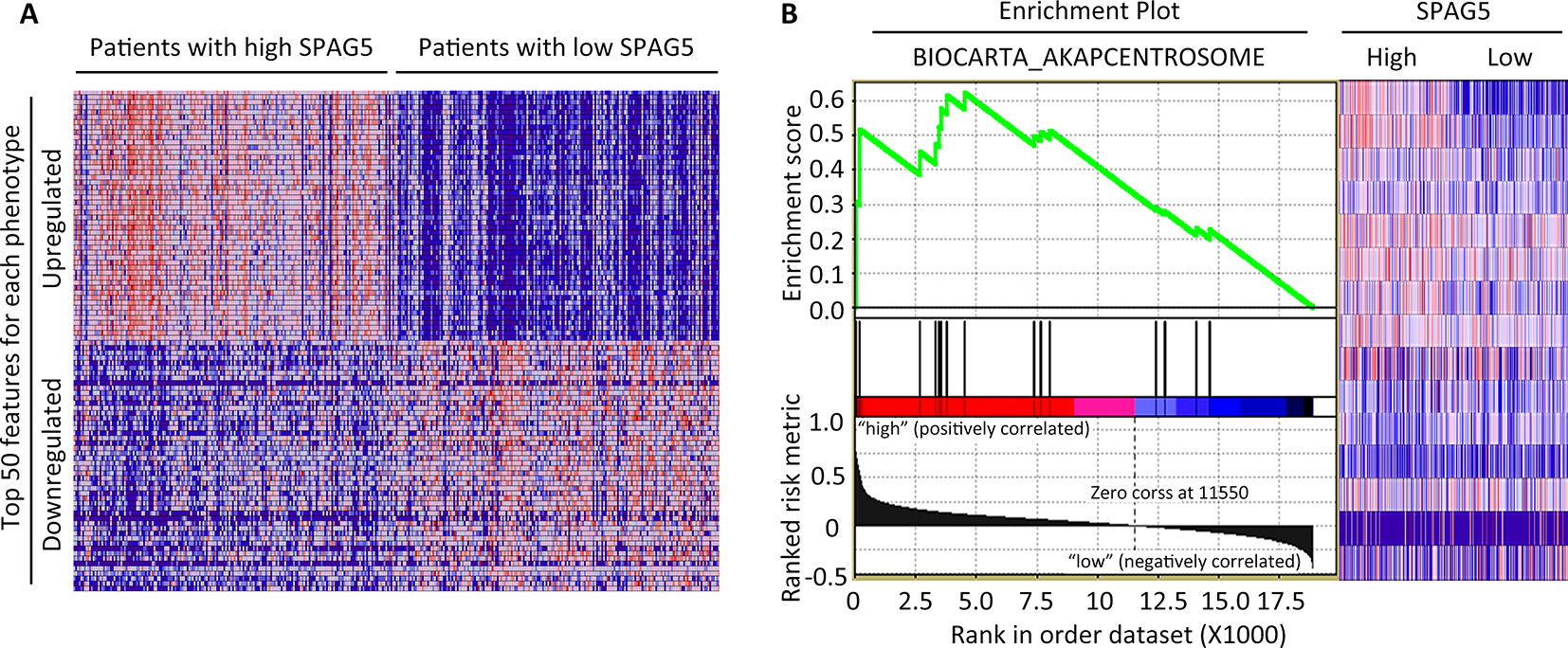

Supplement: Supplementary file 5 — Figure S5. The GSEA analysis of SPAG5 in TCGA data. A. Patients in TCGA dataset were separated into two groups according to the expression of SPAG5. The heatmap showed the top 50 features for each phenotype. B. Gene Set Enrichment Analysis (GSEA) indicated that pathway involved in akapcentrosome was activated in cases with high SPAG5 expression. (JPG 1093 kb) [file 12943_2018_872_MOESM5_ESM.jpg]
